# Sex-related differences in single nucleotide polymorphisms associated with dyslipidemia in a Korean population

**DOI:** 10.1186/s12944-022-01736-5

**Published:** 2022-11-23

**Authors:** Gyeonghee Lee, Hye Kyung Jeon, Hae Young Yoo

**Affiliations:** 1 Department of Nursing, Chung-Ang University, 84 Heukseok-ro, Dongjak-gu, Seoul, 06974 Republic of Korea; 2 Graduate School, Chung-Ang University, Seoul, 06974 Republic of Korea

**Keywords:** Genome-wide association analysis, Dyslipidemia, Sex-related difference, Single nucleotide polymorphism

## Abstract

**Background:**

The prevalence of dyslipidemia has increased steadily in Korea, and the incidence of dyslipidemia differs by sex. In this study, we identified single nucleotide polymorphisms (SNPs) related to dyslipidemia in Korean cohorts through genome-wide association study (GWAS) analysis.

**Methods:**

Genotyping was conducted to determine the genotypes of 72,298 participants and investigate genotypes for 7,079,946 SNPs. Sex, age, and BMI were set as covariates for GWAS, and significant SNPs were identified in the discovery and replication stages using logistic regression.

**Results:**

GWAS of the entire cohort revealed a total of five significant SNPs: rs117026536 *(LPL),* rs651821 *(APOA5),* rs9804646 *(APOA5),* rs9926440 *(CETP),* and rs429358 *(APOE)*. GWAS of the male subjects revealed a total of four significant SNPs. While rs9804646 *(APOA5)* and rs429358 *(APOE)* were significant for all the subjects, rs662799 *(APOA5)* and rs56156922 *(CETP)* were significant only for the male subjects. GWAS of the female subjects revealed two significant SNPs, rs651821 *(APOA5)* and rs9804646 (*APOA5)*, both of which were significant in all the subjects.

**Conclusion:**

This is the first study to identify sex-related differences in genetic polymorphisms in Korean populations with dyslipidemia. Further studies considering environmental variables will be needed to elucidate these sex-related genetic differences in dyslipidemia.

**Supplementary Information:**

The online version contains supplementary material available at 10.1186/s12944-022-01736-5.

## Introduction

Dyslipidemia is defined as abnormal levels of blood lipids such as total cholesterol (TC), low-density lipoprotein cholesterol (LDL-C), high-density lipoprotein cholesterol (HDL-C), and triglycerides (TGs) and is a major cause of the deterioration of vascular function, which results in coronary heart disease (CHD) and stroke [1]. Plasma lipid levels are closely related to traditional CHD risk factors such as age, sex, family history, smoking, hypertension, diabetes mellitus, obesity, and a sedentary lifestyle [2–6]. Furthermore, genetic factors have a considerable influence on lipid metabolism [7]. Therefore, it is recommended that a person with certain risk factors or a family history of dyslipidemia must be screened at a young age [2, 6]. The prevalence of dyslipidemia has continued to increase in recent decades and shows a marked difference between men and women [8, 9]. The Korean Society of Lipid and Atherosclerosis (KSoLA) reported that the prevalence rate of dyslipidemia among Korean adults was 38.4% and was significantly higher in men than in women [8]. In men, dyslipidemia occurred most frequently in men aged 50–59 years and then gradually decreased thereafter; however, in women, the prevalence of dyslipidemia increased rapidly after menopause and exceeded the prevalence in men [10–12]. The rapid increase in the prevalence of dyslipidemia in women could be explained by hormonal changes caused by menopause. Estrogen decreases after menopause, leading to increased LDL-C and decreased HDL-C levels after menopause, especially with a decrease in HDL2-C, which is considered cardioprotective, thus increasing the risk of cardiovascular disease [12]. However, the cause of sex-related differences in the incidence of dyslipidemia has not been elucidated [13].

The levels of LDL-C, HDL-C, and TG interact with each other throughout metabolic processes [14, 15]. Patients are treated for dyslipidemia in the clinic, which takes into account multiple lipid levels rather than the individual lipid levels [10]. In GWAS, however, the interaction of each lipid level with regard to genetic variants was the main focus of SNP identification [16, 17]. Although studies examining the relationships between each lipid level and SNPs in the Korean cohort have been conducted, there have been relatively few that have included dyslipidemia [17]. Considering the metabolic relationship of each lipid level, studies on the association between dyslipidemia and related SNPs are necessary.

In this study, we investigated the genomes of individuals with dyslipidemia through a genome-wide association study (GWAS). This study was based on a method of screening genetic variations significantly affecting clinical or epidemiological variables among numerous single nucleotide polymorphisms (SNPs) present on the SNP chip (SNP microarray chip) [18]. GWAS enables the genetic analysis of individuals to enable the development of targeted preventive strategies for serious disease as well as the provision of individualized medical care [19]. With regard to dyslipidemia, GWAS in the Korean population has thus far been mainly performed using individual lipid levels (TG, TC, HDL-C, and LDL-C) or single gene-related associations; there are few studies on dyslipidemia and none analyzing the genetic association in dyslipidemia with sex [16]. Therefore, in this study, we identified SNPs related to dyslipidemia in a Korean population and identified genetic differences between men and women with dyslipidemia.

## Methods

### Study subjects

The study subjects were selected from the KoGES cohort. To analyze the correlation between dyslipidemia and genetic information with an emphasis on sex-related differences in Koreans, three datasets from the KoGES project were collected from the HEXA, CAVAS, and KARE. In the discovery stage, an urban-based cohort (HEXA) was used for the GWAS analysis. Participants were recruited from Seoul, Busan, Incheon, Daegu, Gwangju, Ulsan, Anyang, Gyeonggi Province, Seongnam, Gangwon, Cheonan, Jeonnam, Hwasun, South Jeolla Province, and Changwon, South Gyeongsang Province, from 2004 to 2013. The analysis included 15,054 dyslipidemia patients and 37,404 controls belonging to the HEXA cohort, with a total of 52,458 participants. Two independent Korean cohorts were used in the replication stage: a rural-based cohort (CAVAS) and a community-based cohort (KARE). The CAVAS cohort was recruited from 2005 to 2011 and comprised 40–69-year-old men and women living in rural areas of Korea: Yangpyeong, Goryeong in Gyeongsangbuk-do, Namwon in Jeonnam, Wonju and Pyeongchang in Gangwon-do, and Ganghwa in Incheon. We used 2743 dyslipidemia patients and 2918 controls in the CAVAS cohort for analysis. The KARE cohort was recruited from 2001 to 2002 and comprised men and women aged 40–69 years living in Ansan and Anseong in Gyeonggi-do. The analysis included 2309 dyslipidemia patients and 3184 controls from the KARE cohort.

### Biochemical measurements

The general and clinical characteristics of the subjects were obtained from data collected through self-report questionnaires and physical examination in the Korean Genome and Epidemiology Study (KoGES). We analyzed sex, age, BMI, TC, LDL-C, HDL-C, and TGs in this study.

According to The Korean Society of Lipid and Arteriosclerosis Korean Guidelines for the management of dyslipidemia, dyslipidemia is defined as one or more cases where TC is > 240 mg/dL, LDL-C is > 160 mg/dL, TGs are > 200 mg/dL, and HDL-C is < 40 mg/dL [10]. Based on this diagnosis criterion, subjects were assigned to the patient group if the results of their fasting serum lipid levels satisfied at least one of the four criteria and to the control group when none of the criteria were met.

### Genotyping and quality control

Genomic DNA samples were genotyped using the Korea Biobank Array (K-Chip), including genetic information from the HEXA, CAVAS, and KARE cohorts, designed by the Korea Centers for Disease Control and Prevention (KNIH, 4845–301, 3000–3031, http://nih.go.kr/NIH_NEW/main.jsp) and manufactured by Affymetrix. In this study, genotyping was conducted to determine the genotypes of 72,298 people and investigate the genotypes for 7,079,946 SNPs. SNP quality control (QC) operations were performed to purify the genomic data, and 5,344,653 SNPs were used after QC by applying minor allele frequency (MAF) < 0.05, SNP call rate < 0.95, and Hardy-Weinberg equal *p* value < 10^− 6^ for practical analysis. A sorting operation was performed to distinguish between the HEXA, CAVAS, and KARE cohorts included in the K-chip. In the GWAS, age, sex, and BMI were set as covariates, and the discovery stage was performed in the HEXA cohort using logistic regression analysis. The analysis of 25,448 out of 58,700 people in the HEXA cohort resulted in a total of 25 significant SNPs. These 25 SNPs were used to perform the replication stage on the CAVAS and KARE cohorts.

### Statistical analysis

Statistical analyses were performed using PLINK (v.1.07, http://pngu.mgh.harvard.edu/purecell/plink/) to derive data refinement and SNPs from the genomic data. FUMA GWAS (https://www.nature.com/articles/s41467-017-01261-5) was used to interpret the data and represent plots [20] and Haploreg v4.1 (HaploReg v4.1 (broadinstitute.org)) for genetic location and in silico analysis. For the functional analysis of significant SNPs revealed through the analysis, dbSNP (https://www.ncbi.nlm.nih.gov/snp/), GWAS Catalog (https://www.ebi.ac.uk/gwas/), and Ensembl (Ensembl genome browser 108) were mainly used. Through these public data, the locus and main functions of the SNPs were analyzed [21].

## Results

### Characteristics of the study subjects

Clinical features related to sex, demographics, and dyslipidemia in the Health EXAminee (HEXA), the cardiovascular disease association study (CAVAS), and the community-based KARE cohorts are described in Table 1. The average age of the subjects was 58.54 years in the HEXA cohort in the discovery stage and 60.64 years and 51.55 years in the CAVAS and KARE cohorts, respectively, in the replication stage. Clinical features associated with dyslipidemia included TC, HDL-C, TG, and LDL-C. According to the Korean Society of Lipid Atherosclerosis Guidelines and ECS/EAS guidelines for dyslipidemia [10, 11], dyslipidemia was defined as one or more of the following four components: TC > 240 mg/dL, LDL-C > 160 mg/dL, TG > 200 mg/dL, and HDL-C < 40 mg/dL. Accordingly, 15,054 cases were included in the dyslipidemia patient group in the discovery stage, and 2743 and 2309 cases in the CAVAS and KARE cohorts were included in the dyslipidemia patient group in the replication stage.

**Table 1:** Characteristics of all the study subjects

Variables	Categories	HEXA(Discovery)			CAVAS(Replication)			KARE(Replication)		
		Dyslipidemia (*n* = 15,054)	Nondyslipidemia (*n* = 37,395)		Dyslipidemia (*n* = 2743)	Nondyslipidemia (*n* = 2912)		Dyslipidemia (*n* = 2309)	Nondyslipidemia (*n* = 3184)	
		n(%) or M ± SD	n(%) or M ± SD	*p*	n(%) or M ± SD	n(%) or M ± SD	*p*	n(%) or M ± SD	n(%) or M ± SD	*p*
**Sex**	Male	5848(38.9)	12,402(33.2)	< 0.001***	1104(40.2)	1043(35.8)	< 0.001***	1303(56.4)	1313(41.2)	< 0.001***
	Female	9206(61.1)	24,993(66.8)		1639(59.8)	1869(64.2)		1006(43.6)	1871(58.8)	
**Age (years)**		58.58 ± 7.77	58.53 ± 8.30	0.087	60.89 ± 8.37	60.40 ± 9.02	< 0.05*	52.21 ± 8.33	51.07 ± 8.60	< 0.001***
**BMI (kg/m**^**2**^)		24.54 ± 2.87	23.62 ± 2.92	< 0.001***	24.91 ± 3.04	23.91 ± 3.16	< 0.001***	25.37 ± 2.80	24.05 ± 2.98	< 0.001***
**TC (mg/dL)**		224.51 ± 45.30	189.19 ± 27.96	< 0.001***	202.25 ± 41.41	189.77 ± 25.84	< 0.001***	213.78 ± 41.49	188.31 ± 26.39	< 0.001***
**HDL-C (mg/dL)**		51.21 ± 16.82	60.72 ± 73.73	< 0.001***	39.40 ± 9.99	51.70 ± 10.10	< 0.001***	43.37 ± 10.50	53.59 ± 10.24	< 0.001***
**TG (mg/dL)**		184.01 ± 112.03	100.94 ± 37.24	< 0.001***	183.18 ± 96.22	107.19 ± 37.10	< 0.001***	216.86 ± 140.55	106.45 ± 38.68	< 0.001***
**LDL-C** **(mg/dL)**		138.84 ± 41.25	108.28 ± 26.08	< 0.001***	127.73 ± 37.74	116.63 ± 24.23	< 0.001***	130.89 ± 38.72	113.44 ± 24.91	< 0.001***

*M* mean, *SD* standard deviation, *P* *p* value, *BMI* body mass index, *TC* total cholesterol, *HDL-C* high-density lipoprotein cholesterol, *TG* triglyceride, *LDL-C* low-density lipoprotein cholesterol, ***p < 0.001, ** *p* < 0.01, * *p* < 0.05

Clinical features related to demographics and dyslipidemia for male subjects in the HEXA (*n* = 18,250), CAVAS (*n* = 2147), and KARE (*n* = 2616) cohorts are described in Additional File 1: Table 1. The average age of the male study subjects was 59.94 years in the HEXA cohort in the discovery stage and 62.13 years and 51.08 years in the CAVAS and KARE cohorts, respectively, in the replication stage. Among the male study subjects, 5848 patients in the HEXA cohort in the discovery stage and 1104 and 1303 in the CAVAS and KARE cohorts in the replication stage, respectively, were included in the dyslipidemia group.

Clinical features related to demographics and dyslipidemia for female subjects in the HEXA (*n* = 34,199), CAVAS (*n* = 3508), and KARE (*n* = 2877) cohorts are described in Additional File 2: Table 2. The average age of the female study subjects was 57.80 years in the HEXA cohort at the discovery stage and 59.72 years and 51.98 years in the CAVAS and KARE cohorts at the replication stage, respectively. Among the female study subjects, 9206 patients from the HEXA cohort at the discovery stage and 1639 and 1006 from the CAVAS and KARE cohorts at the replication stage, respectively, were included in the dyslipidemia group.

### Genome-wide association study of all the subjects

**Fig. 1:**
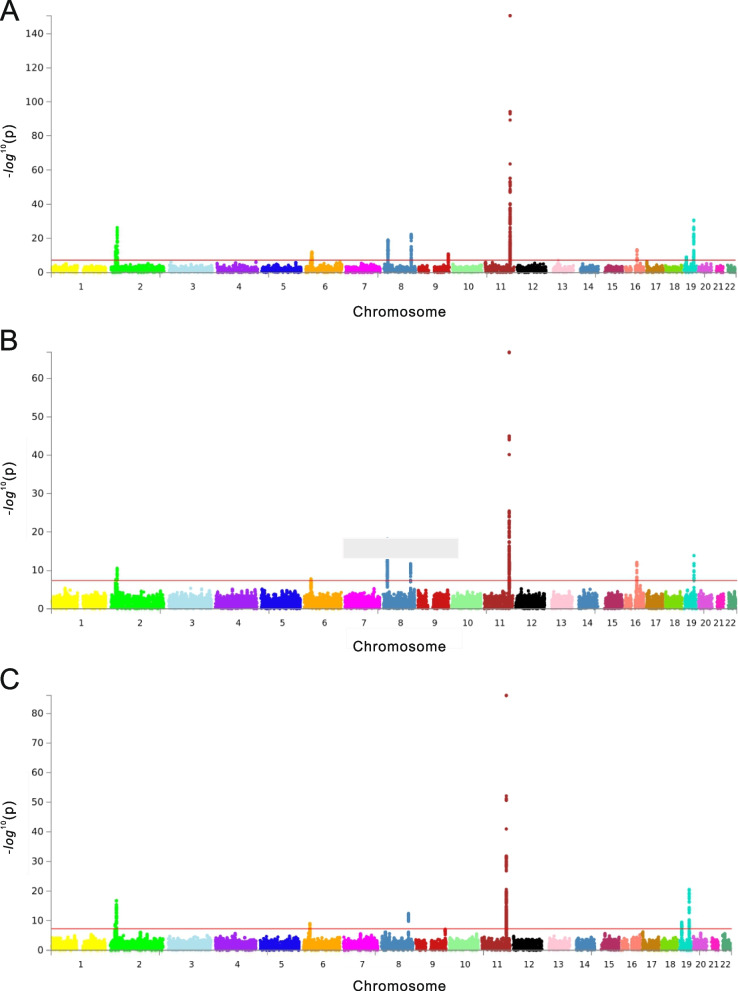
Manhattan plots for the discovery stage.** A** Genome-wide association study (GWAS) analysis of the entire cohort, comprising both men and women, revealed that 25 SNPs in the discovery stage had a significant effect on the incidence of dyslipidemia. **B** GWAS of the male cohorts revealed that 13 SNPs in the discovery stage had a significant effect on the incidence of dyslipidemia. **C** GWAS of the female cohort revealed that 12 SNPs in the discovery stage had a significant effect on the incidence of dyslipidemia. Significant SNPs, with a strict criterion of *p* < 5 × 10^− 8^, were confirmed by Manhattan plots for all the subjects

**Fig. 2:**
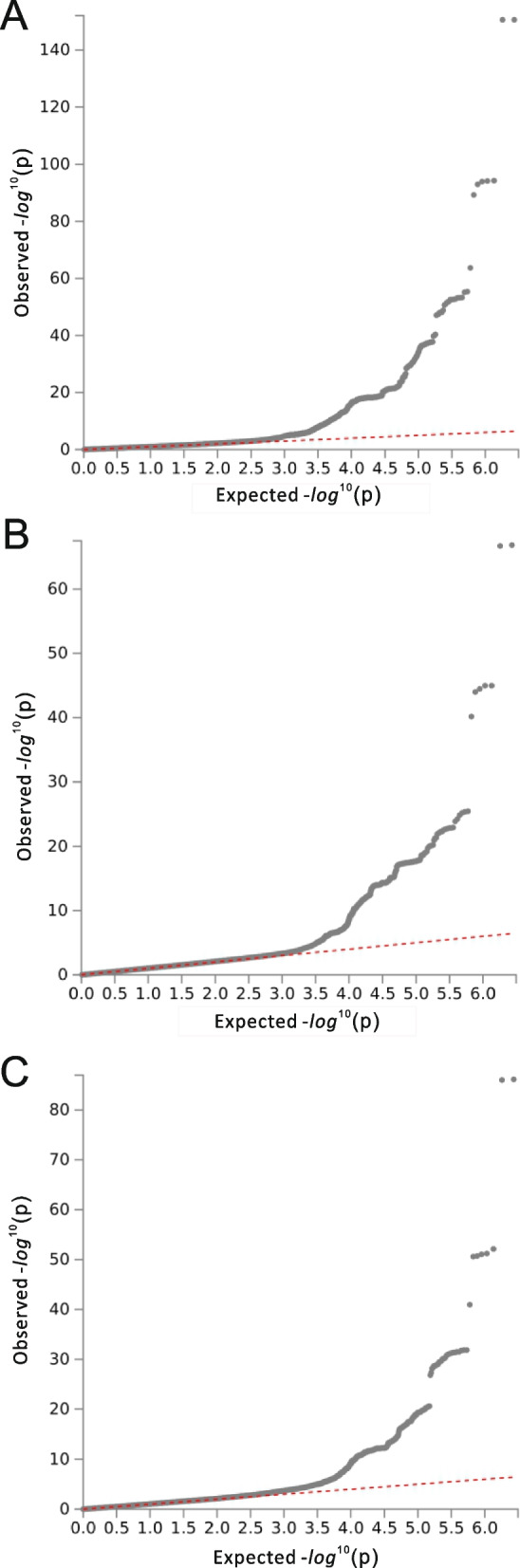
QQ plots for the discovery stage. Some single nucleotide polymorphisms (SNPs) are associated with dyslipidemia in the QQ plots of **A** all cohorts (men and women), **B** male cohort, and **C **female cohort

GWAS analysis of all the subjects revealed 25 significant SNPs in the discovery stage (*P* < 5 × 10^− 8^) (Fig. 1A). It was confirmed through QQ plots that these SNPs were associated with dyslipidemia (Fig. 2A). A total of five significant SNPs were finally discovered in the replication stage that satisfied the Bonferroni-corrected cutoff (*P* < 2 × 10^− 3^). Table 2 shows the GWAS results for dyslipidemia for all the study subjects. The *APOA5* gene rs9804646 was most associated with dyslipidemia (*P* = 1.25 × 10^− 32^); this association was maintained during the replication stage (CAVAS *P* = 1.02 × 10^− 6^, KARE *P* = 2.52 × 10^− 6^). Additionally, rs117026536 in the *LPL* gene, rs651821 in the *APOA5* gene, rs9926440 in the *CETP* gene, and rs429358 in the *APOE* gene were associated with dyslipidemia. The GWAS analysis of the male subjects revealed 13 significant SNPs in the discovery stage (*P* < 5 × 10^− 8^) (Fig. 1B). It was confirmed through QQ plots that these SNPs were associated with dyslipidemia in males (Fig. 2B). In addition, a total of four significant SNPs were discovered in the replication stage that satisfied the Bonferroni-corrected cutoff (*P* < 4.16 × 10^− 3^). Table 3 shows the GWAS results for dyslipidemia in the male subjects. The *APOA5* gene rs662799 was most associated with dyslipidemia (*P* = 1.40 × 10^− 67^); this association was maintained during the replication stage (CAVAS *P* = 3.69 × 10^− 8^, KARE *P* = 1.47 × 10^− 11^). Additionally, rs9804646 in the *APOA5* gene, as well as rs56156922 and rs429358 in the *APOE* gene, were associated with dyslipidemia. Among these SNPs, rs9804646 and rs429358 correspond to the key SNPs found in all the study subjects, while rs56156922 and rs662799 were found only in male subjects. The GWAS analysis of the female subjects revealed 12 significant SNPs in the discovery stage (*P* < 5 × 10^− 8^) (Fig. 1C). It was confirmed through QQ plots that these SNPs were associated with dyslipidemia in females (Fig. 2C). In addition, a total of two crucial SNPs were discovered in the replication stage that satisfied the Bonferroni-corrected cutoff (*P* < 3 × 10^− 3^). Table 4 shows the GWAS results for dyslipidemia in the female subjects. The *APOA5* gene rs9804646 was the most associated with dyslipidemia (*P* = 7.31 × 10^− 87^); this association was maintained during the replication stage (CAVAS *p* = 8.64 × 10^− 4^, KARE *P* = 4.29 × 10^− 4^). Moreover, rs651821 of the *APOA5* gene was also shown to be associated with dyslipidemia. Significant SNPs in the female study subjects were also found in all the study subjects, unlike the significant SNPs found only in the male study subjects. Thus, these results indicate that there is a sex-related association between SNPs and dyslipidemia.

**Table 2:** Significant SNPs associated with dyslipidemia in the entire study

					Discovery					Replication							
					HEXA					CAVAS				KARE			
CHR	POS	SNP	Gene	Location of SNP	Minor allele	MAF	SE	OR	*p*-value	MAF	SE	OR	*p*-value	MAF	SE	OR	*p*-value
8	8:19818773_G/T	rs117026536	LPL	intronic	T	0.1256	0.02137	0.823	7.74E-20	0.1251	0.6964	0.05954	1.24E-09	1.25E-01	0.8262	0.05967	0.001375
11	11:116662579_C/T	rs651821	APOA5	UTR5	C	0.2988	0.01469	1.469	2.70E-15	0.2975	1.441	0.04253	8.61E-18	3.01E-01	1.536	0.04327	3.61E-23
11	11:116665079_C/T	rs9804646	APOA5	intergenic	T	0.1572	0.01952	0.7928	1.25E-32	0.159	0.7745	0.05229	1.02E-06	1.59E-01	0.7725	0.05486	2.52E-06
16	16:57002663_C/G	rs9926440	CETP	intronic	C	0.3132	0.01461	1.101	3.70E-11	0.3159	1.159	0.0411	0.000341	0.3112	1.177	0.04193	0.000104
19	19:45411941_T/C	rs429358	APOE	exonic	C	0.09561	0.02239	1.298	2.14E-31	0.09556	1.366	0.06577	2.09E-06	9.93E-02	1.48	0.06531	1.97E-09

*CHR* chromosome, *POS* position, *SNP* single nucleotide polymorphism, *MAF* minor allele frequency, *SE* standard error, *OR* odds ratio

**Table 3:** Significant SNPs associated with dyslipidemia in the male subjects

Male					Discovery					Replication							
					HEXA					CAVAS				KARE			
CHR	POS	SNP	Gene	Location of SNP	Minor allele	MAF	SE	OR	*p*-value	MAF	SE	OR	*p*-value	MAF	SE	OR	*p*-value
11	11:116663707_G/A	rs662799	APOA5	upstream	G	0.2988	0.0245	1.53	1.40E-67	0.2975	0.0692	1.464	3.69E-08	0.3012	0.06232	1.523	1.47E-11
11	11:116665079_C/T	rs9804646	APOA5	intergenic	T	0.1572	0.03249	0.765	1.64E-16	0.159	0.08539	0.7331	0.000278	0.1589	0.07805	0.7829	0.001717
16	16:56987369_T/C	rs56156922	CETP	intergenic	C	0.1713	0.03093	0.8015	8.36E-13	0.1689	0.08472	0.7201	0.000107	0.1685	0.07429	0.7894	0.001452
19	19:45411941_T/C	rs429358	APOE	exonic	C	0.09561	0.03746	1.334	1.50E-14	0.09556	0.1082	1.416	0.001289	0.09931	0.09677	1.556	4.96E-06

*CHR* chromosome, *POS* position, *SNP* single nucleotide polymorphism, *MAF* minor allele frequency, *SE* standard error, *OR* odds ratio

**Table 4:** Significant SNPs associated with dyslipidemia in the female subjects

Female					Discovery					Replication							
					HEXA					CAVAS				KARE			
CHR	POS	SNP	Gene	Location of SNP	Minor allele	MAF	SE	OR	*p*-value	MAF	SE	OR	*p*-value	MAF	SE	OR	*p*-value
11	11:116662579_C/T	rs651821	APOA5	UTR5	T	0.2988	0.02449	0.8092	5.48E-18	0.2975	0.0544	1.437	2.64E-11	0.3014	0.06172	1.575	1.88E-13
11	11:116665079_C/T	rs9804646	APOA5	intergenic	C	0.1572	0.01844	1.439	7.31E-87	0.159	0.06662	0.8009	0.0008636	0.1589	0.07908	0.7569	0.0004288

*CHR* chromosome, *POS* position, *SNP* single nucleotide polymorphism, *MAF* minor allele frequency, *SE* standard error, *OR* odds ratio

## Discussion

This study attempted to identify genes related to dyslipidemia according to sex in Korean cohorts. We identified five dyslipidemia-related SNPs in the entire study population. We also tried to identify dyslipidemia-related SNPs according to sex by analyzing male and female cohorts separately. In men, four dyslipidemia-related SNPs were discovered, of which two were also found in the entire cohort. Of note, the other two SNPs, rs56156922 (*CETP*) and rs662799 (*APOA5*), appeared to be dyslipidemia-related SNPs found only in men. In contrast, two dyslipidemia-related SNPs were discovered in women, which were also commonly found in the entire cohort. These results confirm that there are sex-related differences in dyslipidemia-related SNPs.

Although rs56156922 located in the *CETP* gene was found to be related to HDL-C levels [22], this is the first report of sex-related differences in this SNP. The *CETP* gene is well known for producing *CETP*, which is a plasma protein that promotes the transport of triglycerides (TGs), exchanging them with cholesterol esters of HDL [23, 24]. Therefore, the *CETP* gene is closely related to TG, LDL-C, and HDL-C. In addition, SNPs that exist at a similar position to rs56156922 in the *CETP* gene show sex-related differences in relation to blood lipid levels. *Taq1B* (rs708272) and the *CETP* polymorphisms C-629A (rs1800775) and I405 V (rs5882) are known to alter blood lipid levels of HDL-C, LDL, and TG [25, 26]. Indeed, rs56156922 and these SNPs are located at chromosome 16q13 within the *CETP* gene, all of which are affected and appear as SNPs associated with blood lipid levels. Previous studies have shown that the *CETP* gene affects lipid levels differentially depending on the sex of the individuals [27–30]. In a study of the association between *CETP* and HDL-C concentrations in Jewish cohorts, the mean plasma *CETP* levels were higher in women than in men, whereas plasma *CETP* levels were inversely proportional to HDL-C in men but not in women [30]. The action of *CETP* generally increases the levels of TGs and LDL-C and lowers the level of HDL-C in a sex-independent manner. However, estrogen has an effect on LDL-C metabolism through LDL receptors and increases HDL-C synthesis by delaying the catabolism of HDL-C [31, 32]. We speculate that estrogen may also play a role in our study. Through the action of estrogen, the activity of *CETP* is reduced, indicating that premenopausal women have higher HDL-C and lower LDL-C concentrations than men or postmenopausal women [33, 34]. It is presumed that this action of *CETP* may have also influenced our results. The SNPs of *CETP*, including rs56156922, may not appear as dyslipidemia-related SNPs in female subjects due to the action of estrogen. Nevertheless, it is difficult to determine whether estrogen has a direct effect on these *CETP* mutations.

Interestingly, rs662799 of *APOA5* also appeared as a dyslipidemia-related SNP only in the male cohort, indicating that there are sex-related differences in the prevalence of dyslipidemia. The *APOA5* protein produced through the *APOA5* gene is the main regulator of plasma triglyceride levels [35]. In the metabolic process of *APOA5*, it activates the metabolism of triglycerides and affects the production of LDL-C and HDL-C by inhibiting the production and assembly of VLDL particles [36]. Specifically, re662799 of APOA5 is known as the SNP, which has a significant association in the Asian population, although it has little effect in the European population [37]. rs662799 of *APOA5* is located at 11q23.3 on chromosome 11. A previous study of blood lipid levels as a major risk factor for CHD showed that rs662799 has a substantial effect on blood lipid levels, with a significant association with CHD in males in particular [38]. Thus, this SNP of *APOA5* could be an important factor in explaining the variations in the incidence of CHD by sex and age, especially the higher incidence of CHD in men than in women due to its sex-specific effects on blood lipid levels [39, 40].

Another SNP of *APOA5*, rs9804646, which is located at 11q23.3, was found to be a dyslipidemia-related SNP in both the male and female cohorts. Although rs9804646 was found to be linked to dyslipidemia in previous studies on East Asian populations [41] and multiethnic populations consisting of East Asians, African Americans, and Hispanics, regardless of country [42], no links have been found in the Korean population. In addition to rs9804646, the SNP rs651821 in the female cohort was also present in *APOA5* and was confirmed to have a significant association with dyslipidemia in the Korean population. Kim et al. showed that rs651821 had a major impact on plasma TG levels in the KoGES’ association analysis with the HEXA and KARE populations [17]. In addition, Gombojav et al. showed that rs6512821 has significant associations with plasma TG levels in the KARE and Healthy Twin Study population of KoGES [43].

rs9926440 of *CETP* was found to be another significant SNP in this study, appearing in the entire cohort. In another study examining various health indicators related to metabolic syndrome, such as blood pressure, blood sugar, and blood lipid levels, rs9926440 was found to be associated with obesity and metabolic syndrome in a Korean cohort [44]. In our study focused on blood lipid levels, rs9926440 was found to be strongly associated with dyslipidemia. Thus, the findings from previous [44] and current studies suggest that rs9926440 is highly correlated with chronic diseases such as metabolic syndrome and dyslipidemia in these Korean cohorts.

The analysis of the entire cohort revealed SNPs located in the *LPL* and *APOE* genes. As the *LPL* gene is associated with high levels of TG and HDL-C, SNPs of this gene would be expected to be found in the entire cohort in this study. The *LPL* gene encodes lipoprotein lipase (*LPL*), which converts VLDL to LDL through catalysis and functions in triglyceride hydrolysis. Mutations that cause *LPL* deficiency are highly associated with hyperlipidemia and various lipoprotein metabolic disorders [45]. Previous studies have shown that rs117026536 in the *LPL* gene was associated with high levels of TG and HDL-C in a non-Hispanic, white American population; however, no studies have been conducted in East Asian populations [46]. This study provides evidence that rs117026536 of the *LPL* gene is related to the occurrence of dyslipidemia in the Korean population. In our study, rs429358 in the *APOE* gene appeared in the male but not in the female subjects. *APOE* is a protein that carries lipids, fat-soluble vitamins and cholesterol into the blood and is produced by the *APOE* gene. Because of its function, it is a protein that is highly related to various lipoprotein metabolism and cardiovascular diseases [47]. Previous studies have shown that rs429358 is linked to HDL-C, LDL-C, TC, and TG levels [48]. Indeed, rs429358 was correlated with TG levels in Korean type 2 diabetes patients [49] and with TG levels and metabolic syndrome in Korean subjects [50]. In addition, rs429358 has been linked to Alzheimer’s disease [51], dementia, and Parkinson’s disease. Of particular relevance to our results, the *APOE* gene has been shown to be associated with androgen, a sex hormone [52, 53], which can explain the sex-related differences in blood lipid levels observed with rs429358, a major SNP determining the genotype of the *APOE* gene.

This study focused on the discovery of SNPs related to dyslipidemia according to sex without including environmental factors or other characteristics. Therefore, a follow-up study is needed to analyze the interactions between environmental factors and dyslipidemia and investigate other sex-related characteristics, such as women’s age and hormone levels. Previous studies have also shown that the effects of diet, smoking, drinking, and physical activity on blood lipid levels and related gene polymorphisms differ between men and women [48]. Pregnant and premenopausal women are less likely to develop dyslipidemia due to the protective effects of estrogen [12]. Menopause reduces HDL-C and increases TG, TC, and LDL-C levels [54]; even healthy, postmenopausal women show increased insulin resistance and body and abdominal fat [55], which are highly linked to blood lipid levels [56]. Therefore, further studies on dyslipidemia and sex-related differences in dyslipidemia are needed.

### Study strengths and limitations

The main strength of this study is that it is the first study conducted on Korean cohorts related to gender differences in dyslipidemia. Furthermore, the cohorts used in this study were organized by the government, making it reliable that the characteristics and biochemical measurements of Koreans were accurately included. Additionally, since three cohorts used in this study also reflect regional features of urban, rural, and community areas in Korea, they can represent dyslipidemia across the entirety of Korea. Finally, it can be considered as a study including the latest trend that needs integrated lipid management rather than each lipid level.

Despite its strengths, this study has several limitations. In this study, a *p* value of 0.05 or less was used when identifying SNPs; however, a p value considering multiple corrections based on different Korean population groups may be more appropriate. In addition, we used blood lipid levels to diagnose the development of dyslipidemia. However, dyslipidemia requires the consideration of all risk factors, such as coronary artery disease, peripheral vascular disease, and diabetes.

## Conclusion

As a result of the genome-wide association study using Korean cohorts, it was confirmed that there is a gender difference in the genetic polymorphisms of dyslipidemia. The findings of this study support that in the treatment of dyslipidemia, both genetic features and gender characteristics should be considered and suggest the direction of customized medical care, which is one of the purposes of the GWAS in clinical practice. On the other hand, since environmental variables should be considered in dyslipidemia, further research is needed including health-related lifestyle factors that make gender differences. In addition, menopause is one of the main causes of specific differences in dyslipidemia that only appear in women compared to men. Therefore, follow-up studies should be conducted considering women’s menopause along with studies including environmental variables.

## Supplementary Information

**Additional file 1.**

**Additional file 2.**

## Data Availability

The data used in this study are available after obtaining permission from the National Biobank of Korea from the National Human Resources Bank of Korea, the Center for Disease Control and Prevention, Republic of Korea.
