# Correction: Sex-related differences in single nucleotide polymorphisms associated with dyslipidemia in a Korean population

**DOI:** 10.1186/s12944-022-01758-z

**Published:** 2022-12-16

**Authors:** Gyeonghee Lee, Hye Kyung Jeon, Hae Young Yoo

**Affiliations:** 1 Department of Nursing, Chung-Ang University, 84 Heukseok-ro, Dongjak-gu, Seoul, 06974 Republic of Korea; 2 Graduate School, Chung-Ang University, Seoul, 06974 Republic of Korea

**Correction: Lipids Health Dis 21, 124 (2022)**

**https://doi.org/10.1186/s12944-022-01736-5**

Following publication of the original article [1], the authors reported an error in acknowledgments. The correct bioresource number in this article's acknowledgments is KBN-2019-066.

The original article has been updated.

## References

[1] Lee G, Jeon HK, Yoo HY (2022). Sex-related differences in single nucleotide polymorphisms associated with dyslipidemia in a Korean population. Lipids Health Dis.

