# Supplementary material for: Sex-related differences in single nucleotide polymorphisms associated with dyslipidemia in a Korean population
Source: Lipids Health Dis. 2022 Nov 23;21:124. doi: 10.1186/s12944-022-01736-5 (PMC9685854; doi:10.1186/s12944-022-01736-5)
Supplement: Supplementary file 1 — Additional file 1. [file 12944_2022_1736_MOESM1_ESM.pdf]

Additional file Table 1. Characteristics of the male study subjects

Variables	HEXA(Discovery)			CAVAS(Replication)			KARE(Replication)		
	**Dyslipidemia (n=5,848)**	**Nondyslipidemia (n=12,402)**		**Dyslipidemia (n=1,104)**	**Nondyslipidemia (n=1,043)**		**Dyslipidemia (n=1,303)**	**Nondyslipidemia (n=1,313)**	
	n(%) or M±SD	n(%) or M±SD	*p*	n(%) or M±SD	n(%) or M±SD	*p*	n(%) or M±SD	n(%) or M±SD	*p*
**Age (years)**	58.74±8.53	60.50±8.54	<0.001***	61.38±8.51	62.89±8.42	<0.001***	50.33±7.81	51.82±8.76	<0.001***
**BMI (kg/m^2)**	25.06±2.64	24.00±2.73	<0.001***	24.85±2.69	23.50±2.91	<0.001***	25.19±2.62	23.69±2.80	<0.001***
**TC (mg/dL)**	207.70±45.75	183.61±28.98	<0.001***	190.51±36.95	185.42±25.99	<0.001***	211.46±40.14	189.04±26.06	<0.001***
**HDL-C (mg/dL)**	44.85±13.37	56.91±12.63	<0.001***	37.24±8.72	51.26±10.27	<0.001***	42.25±9.62	52.60±10.30	<0.001***
**TG (mg/dL)**	211.93±130.17	105.40±38.20	<0.001***	189.01±105.76	107.27±37.56	<0.001***	229.00±148.33	112.07±39.05	<0.001***
**LDL-C (mg/dL)**	123.87±41.65	105.62±27.30	<0.001***	117.18±33.69	112.70±24.76	<0.01**	127.82±37.94	114.03±25.75	<0.001***

M mean, ***SD*** standard deviation, ***P*** *p* value, ***BMI*** body mass index, ***TC*** total cholesterol, ***HDL-C*** high-density lipoprotein cholesterol, ***TG*** triglyceride, ***LDL-C*** low-density lipoprotein cholesterol, ****p*<0.001, ***p*<0.01,

*<*p*<0.05
