# Supplementary material for: Sex-related differences in single nucleotide polymorphisms associated with dyslipidemia in a Korean population
Source: Lipids Health Dis. 2022 Nov 23;21:124. doi: 10.1186/s12944-022-01736-5 (PMC9685854; doi:10.1186/s12944-022-01736-5)
Supplement: Supplementary file 2 — Additional file 2. [file 12944_2022_1736_MOESM2_ESM.pdf]

Additional file Table 2. Characteristics of the female study subjects

Variables	HEXA(Discovery)			CAVAS(Replication)			KARE(Replication)		
	Dyslipidemia (n=9,206)	Nondyslipidemia (n=24,993)		Dyslipidemia (n=1,639)	Nondyslipidemia (n=1,869)		Dyslipidemia (n=1,006)	Nondyslipidemia (n=1,871)	
	n(%) or M±SD	n(%) or M±SD	*p*	n(%) or M±SD	n(%) or M±SD	*p*	n(%) or M±SD	n(%) or M±SD	*p*
**Age (years)**	58.48±7.25	57.55±8.01	<0.001***	60.55±8.25	59.01±9.05	<0.001***	54.65±3.353	50.54±8.45	<0.001***
**BMI (kg/m^2)**	24.21±2.96	23.43±3.00	<0.001***	24.96±3.25	24.13±3.27	<0.001***	25.62±3.03	24.31±3.08	<0.001***
**TC (mg/dL)**	235.19±41.62	191.96±27.03	<0.001***	210.16±42.37	192.19±25.44	<0.001***	216.80±43.01	187.80±26.62	<0.001***
**HDL-C (mg/dL)**	55.25±17.53	62.61±13.85	<0.001***	40.85±10.51	51.94±10.00	<0.001***	44.83±11.39	54.28±10.14	<0.001***
**TG (mg/dL)**	166.26±94.61	98.72±36.55	<0.001***	179.32±89.14	107.15±36.85	<0.001***	201.13±128.17	102.51±37.93	<0.001***
**LDL-C (mg/dL)**	147.97±38.23	109.60±25.35	<0.001***	134.53±38.65	118.82±23.66	<0.001***	134.74±39.35	113.02±24.31	<0.001***

M mean, ***SD*** standard deviation, ***P*** *p* value, ***BMI*** body mass index, ***TC*** total cholesterol, ***HDL-C*** high-density lipoprotein cholesterol, ***TG*** triglyceride, ***LDL-C*** low-density lipoprotein cholesterol, ****p*<0.001, ***p*<0.01,

*<*p*<0.05
